# Impact of National Smoke-Free Legislation on Educational Disparities in Smoke-Free Homes: Findings from the SIDRIAT Longitudinal Study

**DOI:** 10.3390/ijerph120808705

**Published:** 2015-07-24

**Authors:** Giuseppe Gorini, Giulia Carreras, Barbara Cortini, Simona Verdi, Maria Grazia Petronio, Piersante Sestini, Elisabetta Chellini

**Affiliations:** 1 Occupational & Environmental Epidemiology Unit-Cancer Research & Prevention Institute (ISPO), Florence 50141, Italy; E-Mails: g.gorini@ispo.toscana.it (G.G.); b.cortini@ispo.toscana.it (B.C); s.verdi@ispo.toscana.it (S.V.); e.chellini@ispo.toscana.it (E.C.); 2 Prevention Department, Empoli Local Health Authority, Empoli 50053, Italy; E-Mail: mg.petronio@usl11.toscana.it; 3 Section of Phthisiology and Diseases of Respiratory Tract, Siena University, Siena 53100, Italy; E-Mail: sestini@unisi.it

**Keywords:** household smoking bans, socioeconomic, youths

## Abstract

Families with lower socioeconomic status are less likely to adopt household smoking bans (HSB). The aim of this study was to determine whether socioeconomic disparities in HSB prevalence in Italy decreased 7–9 years after the introduction of the Italian ban on smoking in public places. A longitudinal, 12-year, two-wave study was conducted on a sample of 3091 youths aged 6–14 years in 2002; 1763 (57%) were re-interviewed in 2012–2014. A Poisson regression with a robust error variance was used to assess the association between socioeconomic disparities and HSB prevalence. The adoption of HSBs significantly increased from 60% in 2002 to 75% in 2012–2014, with the increase recorded in youths with ≥1 smoking parent only (from 22% at baseline to 46% at follow-up). The presence of HSBs at baseline was more likely in families with ≥1 graduate parent compared to those with no graduate parents (prevalence ratio (PR) = 1.34, 95% confidence interval (CI) = 1.15–1.57), either in families with ≥1 smoking parent (PR = 1.36, 95% CI = 1.17–1.58) or in families with non-smoking parents (PR = 1.61, 95% CI = 1.01–2.56). Conversely, at follow-up socioeconomic disparities dropped since families with no graduate parents were 1.5-fold more likely to introduce a HSB between the two waves. The Italian ban on smoking in public places may have increased the adoption of smoke-free homes in families with smoking and non-graduate parents, causing the drop of the socioeconomic gap in smoke-free homes.

## 1. Introduction

Second-hand smoke (SHS) exposure is known to cause adverse health outcomes among non-smokers [1,2,3,4]. SHS exposure causes lung cancer, coronary heart disease, and stroke in non-smoking adults, exacerbates asthma, and causes impaired lung function, middle ear disease, respiratory illness, and sudden infant death syndrome in children [1,2,3,4].

The World Health Organization Framework Convention on Tobacco Control and its guidelines require ratifying nations to develop smoke-free laws in indoor public places [5,6]. In the last 10 years, clean indoor air laws have been developed in many Countries worldwide [7]. In Italy, the nationwide smoking ban entered into force in 2005 and prohibited smoking in all public places except in locales with a separate smoking room [8]. The implementation of the ban was strongly enforced and was followed by a significant reduction of SHS exposure in hospitality premises [9,10].

Exposure to SHS however still differs markedly between those who live with someone who smokes in the home and those who do not. The prevalence of smoke-free homes has increased as states and communities have enforced legislations on smoke-free public places [11,12]. The adoption of the smoking ban in public places in Italy was not correlated with increased smoking prevalence in private venues (houses and cars) [13]. The ongoing Italian surveillance system for behavioral risk factors (PASSI) reported 78% of respondents living in smoke-free homes in 2012, with a significant 9% increase from 2008 [9]. From 2008 to 2012, a statistically significant increase of smoke-free homes was observed both among smokers (14%) and non-smokers (6%) [9]. 

Low income families and those with less education are less likely to adopt household smoking bans (HSB) [14]. However, there has been limited research on the relationship between HSBs and socioeconomic levels. In two recent longitudinal studies on representative samples of smokers from UK, Australia, USA, and Canada, the presence, introduction, and retention of smoke-free homes increased with increasing socioeconomic status [11,15]. Moreover, immediately after the introduction of the nation-wide smoking ban in public places in 2007, the UK cohort recorded the highest rate of smoke-free home policy introduction [15], indicating that the ban in public places could promote the introduction of a similar ban in smokers’ home. It is well known that socioeconomic disparities in smoking prevalence have widened in Western Countries [16]; therefore, the reduction of social inequalities in smoking and in the adoption of HSBs has become an important public health priority. 

The aim of this paper was to determine whether socioeconomic disparities in HSB prevalence in Italy in a cohort of Tuscan youths decreased 7–9 years after the introduction of the Italian ban on smoking in public places. 

## 2. Experimental Section

### 2.1. First Wave of the Survey (Baseline Survey)

The SIDRIAT (SIDRIA-Italian Studies on Respiratory Disturbances in Childhood and the Environment-in Tuscany) study is a longitudinal, 12-year, two-wave study on a sample of Tuscan youths aged 6–14 years in 2002 who were enrolled in the SIDRIA-2 study. 

The SIDRIA-1 and 2 cross-sectional surveys, two Italian extensions of the ISAAC studies, were aimed at evaluating the prevalence of respiratory and allergic disorders, and their geographic and temporal variations. [17,18,19] The second survey (SIDRIA-2) was performed in 2002 on a representative sample by age and gender of school children aged 6–7 years and adolescents aged 13–14 years in 13 Italian areas. Information on the cohort of school children aged 6–7 years old was collected through a questionnaire completed at home by their parents, whereas figures on the cohort of adolescents were collected using two questionnaires, one completed at home by their parents, and one completed at school by the adolescents themselves.

**Figure 1:**
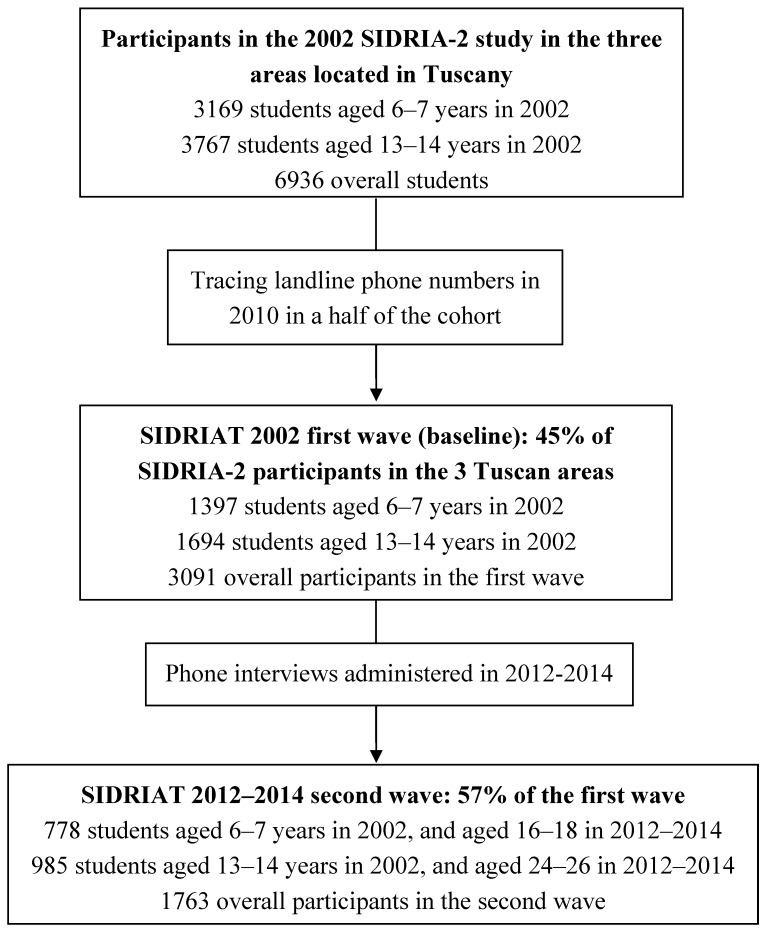
Flow chart of the SIDRIA-Italian Studies on Respiratory Disturbances in Childhood and the Environment-in Tuscany (SIDRIAT) study.

The present SIDRIAT study was conducted in the three SIDRIA-2 study areas located in Tuscany (Florence, Empoli, Siena; 1.25 millions of total inhabitants in 2002). Information regarding youths’ and parents’ smoking status, parents’ educational level, and household smoking ban prevalence was collected for 3,169 children aged 6–7 years and 3767 adolescents aged 13–14 in 2002 (6936 youths, in total) (Figure 1). For the cohort of children, variables used in the SIDRIAT study (parental smoking status and educational level, presence of the HSB) were taken from the questionnaires completed by their parents. For the cohort of adolescents information regarding youths’ and parents’ smoking status, and presence of HSBs was taken from the questionnaires completed by adolescents themselves, whereas parental educational level was taken from the questionnaires completed by their parents. In 2010, due to temporal and funding constraints we decided to trace about a half of the baseline cohort, so we traced landline phone numbers of 3091 youths who were equally distributed in the three areas (44.6% of the initial SIDRIA-2 youths): 1397 and 1694 from the younger and older cohorts, respectively, and these became the first-wave baseline cohort (Figure 1). Considering gender and age, the two variables used for estimating SIDRIA-2 representative sample, there were no differences between SIDRIAT participants (N = 3091) and those with no traced phone numbers (N = 3845). 

### 2.2. Second Wave (Follow-Up Survey)

From December 2011 to April 2014 1763 phone interviews were conducted with 778 adolescents aged 16–18, who were children aged 6–7 in 2002, and with 985 young adults aged 24–26, who were adolescents aged 13–14 in 2002 (Figure 1). The follow-up rate was 57.0% (1763/3091 youths); 12.6% refused to participate to the follow-up; 28.2% had inactive or non-existent phone numbers, and 14.9% of the sample was not possible to contact and interview. Phone interviews were administered by a trained health advisor using a standardized questionnaire on youths’ smoking habits, perceived prevalence of youth and adult smoking, perceived social acceptability of smoking, household smoking ban, and smoking status of parents, siblings, and best friends. Young adults and parents of minors provided informed consent during the calls. The SIDRIAT protocol was approved by the Ethics Committee of the Local Health Authority of Florence in May 2010.

A comparison between the follow-up respondents (1763) and non-respondents (1328 youths) was carried out on the main individual characteristics (gender, sex, and parents’ education): 58% and 42% of interviewed and not interviewed youths, respectively, had ≥1 graduate parent (*p* < 0.001), whereas no differences were found for age and gender.

Only 53 of interviewed young adults (5.4%) no longer lived with their parents at the time of interviews. This is a common phenomenon in Italy [20]. Analyses excluding the 53 young adults not living with their parents at follow-up did not significantly change the results.

### 2.3. Study Variables

Parents’ education at baseline (at least one graduate parent *versus* no graduate parents) was used in order to assess socioeconomic variations in prevalence of smoke-free homes. Using educational level as an indicator of socioeconomic status has the double advantage of being stable over the study period, and being easily and accurately recorded.

Youths were classified as living in a smoke-free home at the baseline survey if both parents were non-smokers or they did not smoke anywhere at home, and at the follow-up survey if youths reported that guests and family members were not allowed to smoke anywhere at home. Thus, it was possible to define four groups: best homes, *i.e.*, those who reported to live in smoke-free environments in both surveys; improving homes: *i.e.*, those who declared a HSB at follow-up but not at baseline; worsening homes, *i.e.*, those who reported a HSB at baseline but not at follow-up; worst homes, *i.e.*, those who declared to live in smoke-polluted environments in both surveys.

Both surveys recorded whether youths had at least one smoking parent. Additionally, in the follow-up survey we collected smoking status of siblings and best friends. Thus, it was possible to define four groups depending on parents’ smoking status at baseline and follow-up. 

### 2.4. Data Analysis

It is usually preferable to estimate prevalence ratios (PRs) instead of odds ratios in cross-sectional studies when conditions are not rare, the prevalence is higher than 10% [21], in order to avoid the overestimation of the effect due to the high prevalence. Thus, in order to assess socioeconomic variations in the prevalence of smoke-free homes that were more 20% in all analyses a multi-varied Poisson regression model was applied with a robust error variance adjusting for gender, cohort, age, youth’s smoking status at baseline, and parental smoking [22]. In order to avoid the potential collinearity between socioeconomic status and the parents’ smoking status the same regression model was conducted stratified by parents’ smoking status. Moreover an analysis considering siblings’ and best friends’ smoking status at follow-up was carried out.

## 3. Results and Discussion

### 3.1. Baseline Characteristics

Table 1 shows the characteristics of participants in both waves by HSB prevalence and parents’ smoking status at baseline. Among the 1763 participants, 42% (=736/1763) reported to have at least one smoking parent at baseline, and 22% of them (=161/736) reported HSBs. Youths with non-smoking parents at baseline were 58% of respondents (=1027/1763), and 88% of them (=905/1027) had HSBs (Table 1). Youths with HSBs at baseline were more likely to have ≥1 graduate parent (26% = 224 + 50/905 + 161 = 274/1066) compared to those without smoke-free homes (13% = 17 + 72/101 + 570 = 89/671; Table 1). Thirty-two percent of fathers and 22% of mothers were smokers at baseline, in line with 2002 figures of smoking prevalence of Italian men and women of the same age-groups of SIDRIAT parents. In fact, smoking prevalence in Italian men aged 35–55 years and in Italian women aged 25–45 years in 2002 were 37% and 22%, respectively [23]. 

**Table 1:** Characteristics of participants in both waves (N = 1763) by household smoking ban (HSB) prevalence and parents’ smoking status at baseline.

	Non-Smoking Parents (N = 1027*)		≥1 Smoking Parent (N = 736*)	
	HSB (N = 905) n (%)	No HSB (N = 101) n (%)	HSB (N = 161) n (%)	No HSB (N = 570) n (%)
**Gender, Boys**	460 (50.8)	43 (42.6)	66 (41.0)	301 (52.8)
**Cohort, Children**	443 (49.0)	12 (11.9)	96 (59.6)	218 (38.3)
**Smoking Status, Current Smoker**	4 (0.4)	13 (12.9)	2 (1.2)	21 (3.7)
**Parents’ Educational Level, ≥1 Graduate Parent**	224 (24.8)	17 (16.8)	50 (31.1)	72 (12.6)

* For 21 youths with non-smoking parents and for 5 youths with ≥1smoking parent figures on household smoking ban were not available.

### 3.2. Trends in HSB

Youths reporting HSBs significantly increased from 60% at baseline (=1066/1763) to 75% at follow-up (1323/1763; *p* < 0.001). The prevalence of HSBs doubled in youths with at least one smoking parent, whereas it did not significantly change in youths with non-smoking parents. In fact, at baseline 22% of youths with at least one smoking parent at baseline (=161/736; Table 1) lived in smoke-free homes, whereas at follow-up 46% of youths with at least one smoking parent at follow-up (=185/400; *p* < 0.001) reported HSBs. In contrast, at baseline 88% of youths with non-smoking parents at baseline (=908/1027) lived in smoke-free homes, and at follow-up 1,138 out of 1,363 youths (84%) living with non-smoking parents at follow-up, recorded HSBs. At follow-up only 16% of fathers and 13% of mothers were smokers. In fact, during the study period about 51% of smoking fathers at baseline and 41% of smoking mothers at baseline successfully quitted. In particular, 55% and 44% of non-graduate and smoking fathers and mothers, respectively, successfully quitted during the study period, whereas graduate parents were less likely to stop during the study period (26% for fathers and 33% for mothers). Figures of 2013 smoking prevalence in Italian men aged 45–64 years and women aged 35–54 years were significantly higher (30% and 20%, respectively) [23] than those reported in SIDRIAT parents.

### 3.3. Socioeconomic Status and Presence, Adoption and Removal of A HSB

The presence of HSBs at baseline was more likely in families with at least one graduate parent compared to those with no graduate parents (Prevalence Ratio [PR] = 1.34, 95% confidence interval [CI] = 1.15–1.57; Table 2), either in families with at least one smoking parent (PR = 1.36, 95% CI = 1.17–1.58) or in families with non-smoking parents (PR = 1.61, 95% CI = 1.01–2.56).

**Table 2:** Prevalence of home smoking ban (HSB) at baseline and follow-up by parents’ educational level, prevalence ratios (PRs) and corresponding 95% confidence intervals (CI) for the association of parents’ educational level and household smoking ban at baseline or follow-up. HSB: household smoking ban.

	**Baseline**		
	**Absence of HSB N (%)**	**Presence of HSB N (%)**	**Presence *vs.* Absence of HSB PR (95%CI) ^§^**
**No Graduate Parents**	525 (78.2)	745 (69.9)	1*
At Least One Graduate Parent ^**^	89 (13.3)	274 (25.7)	1.34 (1.15–1.57)
	**Follow**-**Up**		
	**Absence of HSB N (%)**	**Presence of HSB N (%)**	**Presence *vs.* Absence of HSB PR (95%CI) ^§§^**
**No Graduate Parents**	298 (72.2)	993 (73.7)	1*
At Least One Graduate Parent ^**^	83 (20.1)	283 (21.0)	0.98 (0.80–1.19)

^*^ Reference category; ^**^ Figures on parents’ educational levels were not available for 104; ^§^ Adjusted for gender, cohort, youths’ smoking status at baseline, presence of at least 1 smoking parent at baseline; ^§§^ Adjusted for gender, cohort, youths’ smoking status at baseline, presence of at least 1 smoking parent at follow-up.

**Table 3:** Prevalence of best homes, improving homes, worsening homes, worst homes by parents’ educational level, prevalence ratios (PRs) and corresponding 95% confidence intervals (CI) for the association of parents’ educational level and the change in household smoking ban from baseline to follow-up. Best homes: home smoking ban at both surveys; Improving homes: absence of a home smoking ban at baseline and presence at the follow-up; Worst homes: no smoking ban at both surveys; Worsening homes: presence of a home smoking ban at baseline and absence at follow-up.

	**Best Homes N (%)**	**Improving Homes N (%)**	**Worsening Homes N (%)**	**Worst Homes N (%)**	**Best + Worsening + Worst Homes N (%)**
**At Least One Graduate Parent**	229 (25.1)	50 (12.1)	44 (28.8)	39 (15.1)	324 (23.6)
**No Graduate Parents**	645 (70.8)	327 (79.4)	99 (64.7)	198 (76.4)	976 (71.0)
		**Improving *vs*. Best Homes, PR (95% CI) ^§^**	**Worsening *vs*. Best Homes, PR (95% CI) ^§^**	**Worst *vs.* Best Homes PR (95% CI) ^§^**	**Improving *vs.* All Other Homes PR (95% CI) ^§^**
**At Least One Graduate Parent**		1 ^*^	1 ^*^	1 ^*^	1 ^*^
**No Graduate Parents**		1.49 (1.19–1.88)	0.88 (0.64–1.23)	1.43 (1.14–1.81)	1.48 (1.15–1.92)

^*^ Reference category; ^§^ Adjusted for gender, cohort, youths’ smoking status at baseline, presence of at least 1 smoking parent at baseline and at follow-up.

At follow-up smoke-free homes were equally distributed between families with and without graduate parents (PR = 0.98 95% CI = 0.80–1.19), either in families with or without smoking parents (PR = 1.14, 95% CI = 0.89–1.46; PR = 0.91, 95% CI = 0.66–1.25, respectively). 

Moreover, in between the two surveys, improving homes, *i.e.*, those with HSB introduction in the study period, in comparison with best homes, *i.e.*, those with HSBs in both waves, or in comparison to all other types of homes (best, worsening, and worst homes) were 1.5-fold more likely to belong to families with no graduate parents (PR = 1.49, 95% CI = 1.19–1.88 compared to best homes; PR = 1.48, 95% CI = 1.15–1.92 compared to all other homes; Table 3). 

The stratified analyses by parents’ smoking habits at baseline and follow-up showed that in comparison with best homes, improving homes were more likely to belong to families with no graduate parents who quitted smoking during the study period (*i.e.*, youths with at least one smoking parent at baseline and no smoking parents at follow-up; PR = 1.64, 95% CI = 1.20–2.25) and to parents who did not stop smoking during the study period (*i.e.*, youths with at least one smoking parent at both waves; PR = 1.64, 95% CI = 1.10–2.42). In comparison with all other types of homes, improving homes were more likely to belong to families with parents who quitted smoking between the two waves (PR = 1.77, 95% CI = 1.24–2.53). 

At follow-up, worst homes were still more frequent in families with no graduate parents. In fact, in comparison with best homes, worst homes were1.5-fold more likely to belong to families with no graduate parents (PR = 1.43, 95% CI = 1.14–1.81). This was due to smoking parents at both waves (*i.e*., those who did not quit during the study period; PR = 1.31, 95% CI = 1.03–1.66).

Results from the analyses considering smoking status at follow-up of siblings and best friends did not significantly change (data not shown).

### 3.4. Discussion

Youths reporting HSBs significantly increased from 60% in 2002 to 75% in 2012–2014. This was similar to what has been reported in other Countries after the introduction of a nation-wide smoking ban. [19] The increase, from 22% to 46%, occurred in families with at least one smoking parent. More than a half of bans at follow-up in homes with no HSBs at baseline, *i.e.* improving homes, was associated with parents’ quitting smoking. 

Socioeconomic disparities in HSBs between 2002 and 2012–2014 dropped. In between the two surveys, in comparison to all other types of homes (best, worsening, and worst homes), improving homes were over 1.5-fold more likely to belong to families with no graduate parents, either in families with parents who quitted smoking between the two waves or in families with parents who continued smoking during the study period. Thus, the adoption of HSBs in low socio-economic status households occurred for two concomitant phenomena: not only because parents quitted during the study period, but also because in other low socio-economic status families parents who continued smoking, began doing so outside the home.

At baseline, the socioeconomic gap in smoke-free homes was in line with that observed in previous studies, in which low parental education as well as other socioeconomic indicators, e.g., unemployment, low income, family size, were associated with SHS exposure of children at home [11,15,24,25].

An increase in HSB prevalence was also recorded after the introduction of the nation-wide smoking ban, as it was in two longitudinal studies on cohorts of smokers with one-year follow-up in which, however, the socioeconomic gap in the HSB prevalence remained unchanged after the smoking ban in public places [11,15]. 

In contrast, in this study, characterized by a longer follow-up, the socioeconomic gap dropped even in families with smokers, suggesting that the 2005 smoking ban in public places in Italy not only increased the adoption of HSB, but also contributed to reduce the socioeconomic gap in its promotion of smoke-free homes. 

Therefore, an important public health policy implication of this study is that nation-wide bans on smoking in public places, such as the one introduced in Italy in 2005, may promote the adoption of HSBs in families with lower educational level and in families with smokers. Thus, in order to reduce the socio-economic gap in the adoption of smoke-free homes, the public health recommendation is to implement nation-wide smoking bans, a low-cost policy that not only reduces SHS exposure in public places, but also may foster the adoption of smoking bans in private settings.

Despite these implications, this study has some limitations. First, the SIDRIAT baseline cohort was almost the half of the representative sample interviewed for the SIDRIA-2 study, even though SIDRIAT participants had a similar distribution for age and gender, the variables used to estimate the youths’ representative sample in the SIDRIA-2 study. As a result, parental smoking prevalence at baseline was in line with the smoking prevalence recorded in Italy in 2002, suggesting that the baseline sample was representative of the Italian population. Moreover, more than a third of the study participants was not interviewed, and interviewed participants were more likely to belong to families with at least one graduate parent. As a result, parental smoking prevalence recorded at follow-up was significantly lower than that recorded in Italian men and women of the same age in 2013, given that quitting smoking is more likely to occur in families with more educated parents [26]. A third limitation is that all figures were based on self-report. A fourth limitation is that in the first wave all figures were assessed using a self-administered questionnaire, whereas in the second wave figures were assessed using a phone interviews conducted by a trained health advisor. 

## 4. Conclusions

In conclusion, the Italian ban on smoking in public places may have increased the adoption of smoke-free homes even in families with smoking and non-graduate parents, causing a reduction of the socioeconomic gap in the implementation of smoke-free homes.

## References

[1] US Department of Health and Human Services (2006). The Health Consequences of Involuntary Exposure to Tobacco Smoke: A Report of the Surgeon General.

[2] International Agency for Research on Cancer (2009). Evaluating the effectiveness of smoke-free policies. Handbooks of Cancer Prevention, Tobacco Control.

[3] US Department of Health and Human Services (2014). The Health Consequences of Smoking—50 Years of Progress: A Report of the Surgeon General.

[4] Oberg M., Jaakkola M.S., Woodward A., Peruga A., Prüss-Ustün A. (2011). Worldwide burden of disease from exposure to second-hand smoke: A retrospective analysis of data from 192 countries. Lancet.

[5] World Health Organization (WHO) (2003). WHO Framework Convention On Tobacco Control.

[6] World Health Organization (WHO) (2013). WHO Framework Convention on Tobacco Control: Guidelines for Implementation. http://apps.who.int/iris/bitstream/10665/80510/1/9789241505185_eng.pdf?ua=1.

[7] (2009). WHO Report on the global tobacco epidemic. Implementing Smoke-Free Environments.

[8] (2013). WHO Report on the global tobacco epidemic. Enforcing Bans on Tobacco Advertising, Promotion and Sponsorship.

[9] Minardi V., Gorini G., Carreras G., Masocco M., Ferrante G., Possenti V., Quarchioni E., Spizzichino L., Galeone D., Vasselli S., Salmaso S. (2014). Compliance with the smoking ban in Italy 8 years after its application. Int. J. Public Health.

[10] Gorini G., Moshammer H., Sbrogiò L., Gasparrini A., Nebot M., Neuberger M., Tamang E., Lopez M.J., Galeone D., Serrahima D. (2008). Second-hand smoke Exposure in Italian and Austrian Hospitality premises before and after 2 years from the introduction of the Italian smoking ban. Indoor Air.

[11] Borland R., Yong H.H., Cummings K.M., Hyland A., Anderson S., Fong G.T. (2006). Determinants and consequences of smoke-free homes: findings from the international tobacco control (ITC) Four Country Survey. Tob. Control.

[12] Ferketich A.K., Lugo A., La Vecchia C., Fernandez E., Boffetta P., Clancy L., Gallus S. (2014). Relation between national-level tobacco control policies and individual-level voluntary home smoking bans in Europe. Tob. Control.

[13] Martínez-Sánchez J.M., Blanch C., Fu M., Gallus S., La Vecchia C., Fernández E. (2014). Do smoke-free policies in work and public places increase smoking in private venues?. Tob. Control.

[14] Mills A.L., White M.M., Pierce J.P., Messer K. (2011). Home smoking bans among US households with children and smokers: opportunities for intervention. Am. J. Prev. Med..

[15] King B.A., Hyland A.J., Borland R., McNeill A., Cummings K.M. (2011). Socioeconomic variation in the prevalence, introduction, retention, and removal of smoke-free policies among smokers: Findings from the International Tobacco Control (ITC) Four Country Survey. Int. J. Environ. Res. Public Health.

[16] Huisman M., Kunst A.E., Mackenbach J.P. (2005). Educational inequalities in smoking among men and women aged 16 years and older in 11 European countries. Tob. Control.

[17] Pearce N., Aït-Khaled N., Beasley R., Mallol J., Keil U., Mitchell E., Robertson C., ISAAC Phase Three Study Group (2007). Worldwide trends in the prevalence of asthma symptoms: Phase III of the International Study of Asthma and Allergies in Childhood (ISAAC). Thorax.

[18] Galassi C., De Sario M., Biggeri A., Bisanti L., Chellini E., Ciccone G., Petronio M.G., Piffer S., Sestini P., Rusconi F., Viegi G., Forastiere F. (2006). Changes in prevalence of asthma and allergies among children and adolescents in Italy: 1994–2002. Pediatrics.

[19] Galassi C., Forastiere F., Biggeri A., Gabellini C., De Sario M., Ciccone G., Biocca M., Bisanti L., Gruppo Collaborativo SIDRIA-2 (2005). SIDRIA second phase: Objectives, study design and methods. Epidemiol. Prev..

[20] Sestito L.A., Sica L.S. (2014). Identity formation of Italian emerging adults living with parents: A narrative study. J. Adolesc..

[21] Petersen M.R., Deddens J.A. (2008). A comparison of two methods for estimating prevalence ratios. BMC Med. Res. Methodol..

[22] Zou G. (2004). A Modified Poisson Regression Approach to Prospective Studies with Binary Data. Am. J. Epidemiol..

[23] Italian National Institute of Statistics (ISTAT) Warehouse of statistics currently produced by ISTAT. http://dati.istat.it/.

[24] Mons U., Nagelhout G.E., Allwright S., Guignard R., van den Putte B., Willemsen M.C. (2012). Impact of national smoke-free legislation on home smoking bans: Findings from the International Tobacco Control Policy Evaluation Project Europe Surveys. Tob. Control.

[25] Bolte G., Fromme H. (2008). Socioeconomic determinants of children’s environmental tobacco smoke exposure and family’s home smoking policy. Eur. J. Public Health.

[26] Zhuang Y.L., Gamst A.C., Cummins S.E., Wolfson T., Zhu S.H. (2015). Comparison of smoking cessation between education groups: findings from 2 US National Surveys over 2 decades. Am. J. Public Health.

